# Identification of the antigen recognized by rHIgM22, a remyelination-promoting human monoclonal antibody

**DOI:** 10.1186/2193-1801-4-S1-P15

**Published:** 2015-06-12

**Authors:** Sara Grassi, Simona Prioni, Yana Zorina, Livia Cabitta, Sandro Sonnino, Alessandro Prinetti

**Affiliations:** Department of Medical Biotechnology and Translational Medicine, University of Milano, Italy; Acorda Therapeutics, Inc, Ardsley, USA

**Keywords:** multiple sclerosis, remyelination, sulfatide

Recombinant human IgM22 (rHIgM22) binds to myelin and to oligodendrocytes, and promotes remyelination in a mouse model of multiple sclerosis [1]. rHIgM22 preferentially reacts with sulfatide-positive (O4-positive) oligodendrocytes [1]. Moreover, binding of rHIgM22 is abolished in CNS tissue slices from Cst(-/-) mice [2], suggesting that its binding to myelin requires the presence of a product of cerebroside sulfotransferase, possibly sulfatide, abundantly expressed in oligodendrocytes and in myelin. However the exact identity of the antigen recognized by this antibody remains to be elucidated. We have tested the binding of rHIgM22 to purified lipids and to lipid extracts prepared from mouse brain, brain myelin, mixed glial cultures, and O4-positive oligodendrocytes using TLC immunostaining and ELISA using liposomes and lipid monolayers with different composition. Our preliminary results show that rHIgM22 binds to sulfatide in vitro, while it does not bind to other myelin sphingolipids, including galactosylceramide and sphingomyelin, suggesting that sulfatide at the oligodendrocyte surface might be important for the binding of rHIgM22 to the surface of these cells and to myelin. However, IgM22 does not bind structures expressing sulfatide outside the nervous system, thus additional factors are likely relevant for the immunoreactivity of IgM22 in CNS. Indeed, we have observed in lipid extracts from different sources another lipid molecule selectively recognized by rHIgM22, whose identity is still under investigation. Remarkably, this lipid is also present in the extracts from mixed glial cultures, which do not contain mature O4-positive oligodendrocytes, suggesting that other glial cells in addition to oligodendrocytes might be important in the response to rHIgM22.
